# Detection of Changes of Ancient Buildings from Terrestrial Laser Scanning and Hyperspectral Imaging

**DOI:** 10.1155/2021/3760592

**Published:** 2021-09-18

**Authors:** Xiao Zhang, Rongqing Ma, Ruoyi Gao

**Affiliations:** College of Art, Taiyuan University of Technology, Jinzhong 030600, China

## Abstract

Ancient buildings have various geometric and material changes caused by the historical and natural factors, and their comprehensive detection has also been a more important challenge. This way, in this paper, a flexible, scientific approach from terrestrial laser scanning and hyperspectral imaging is provided for this issue. It is possible to flexibly and accurately detect some potential crisis, which cannot be found in some surface phenomena of historical buildings. Furthermore, one of the main characteristic of this method is that the time and place of the two data acquisition need not be limited, but they can be accurately fused. Another one of the main features is that the fusion data can synthetically detect geometric and material changes of historical buildings. This method was applied to the case study of the Beijing Tianningsi Tower, an extremely dazzling pearl of the Chinese Buddhist pagoda, on which the signs of deformation and restoration were found in the tower shape and in the tower-body sculpture. It was possible to assess the typical physical, chemical, and biological changes of historical buildings, to provide scientific basis for comprehensive research. The results demonstrate that this method is feasible and applicable for detecting changes of ancient buildings and is applied to similar research using more analytical methods for multisource data.

## 1. Introduction

The research of conservation, restoration, and humanities of ancient buildings has been an important topic for many years, specially detecting and quantifying the geometric deformation, material deterioration, and microbial growth mildew of ancient buildings caused by natural or human factors, which is helpful to further evaluate their status and plan possible intervention measures in the future [1, 2]. In recent years, due to their high flexibility and no damage to buildings compared with classical systems, the contactless and noninvasive techniques, such as terrestrial laser scanning [3, 4], infrared thermography (IRT) [5, 6], and Multispectral or Hyperspectral Technique [7, 8], were used to detected historical buildings. Particularly, the different types of terrestrial laser scanner comparing historical objects and infrared thermography detecting of building diagnostics have accelerated the protection and restoration of cultural heritage [9].

However, the changes of ancient buildings are usually caused by geometric, biological, chemical, and other reasons. In addition, some buildings have been repaired many times in history, and the building materials are different, although the surface observation is the same. Single technology can not accurately detect these changes, such as laser scanning detecting geometric deformation and digital images detecting color changes. If we use multiple devices and the integration of multiple technologies can extract more information from multiple angles, we can observe historical buildings more effectively [1]. At present, the most popular fusion method is the fusion of TLS and digital imaging. For example, Tangible cultural heritage is assessed by Point Cloud and Background Photographic Image [10]. And cultural heritage is surveyed to create a 3D multiscale database based on image and active sensor [11]. TLS data with digital images strengthen the recognition of interesting details and their precise geometrical localization in the space because of the high-resolution image information [12, 13].

Compared with digital images having only three band spectral information like red, green, and blue, the hyperspectral data have hundreds or even thousands of band spectral information, which can detect quickly light frequencies and relative intensities of building surface. However, the fusion of laser scanning and hyperspectral imaging is rarely used, due to the so complicated fusion method caused by the different sensors and imaging modalities. This paper proposes a method to detect changes of ancient buildings using laser scanning and hyperspectral imaging. That is, laser data and hyperspectral data are first fused, and then they are used to comprehensively and accurately detect and analyze the change and history of ancient buildings. In the fusion data, the laser information detected the geometric change like incline, convex, and concave by point cloud 3D coordinates, and the hyperspectral information detects the material change caused by corroding, weathering, and salt blooming. This method was applied to the case study of Tianningsi Tower in Beijing, China.

## 2. Materials and Methods

### 2.1. Data Acquisition Equipment

In this work, data acquisition depends on the terrestrial laser scanning system and the terrestrial sweep hyperspectral scanning system, respectively. In either the data acquisition time or location, these two systems work independently.

**Figure 1:**
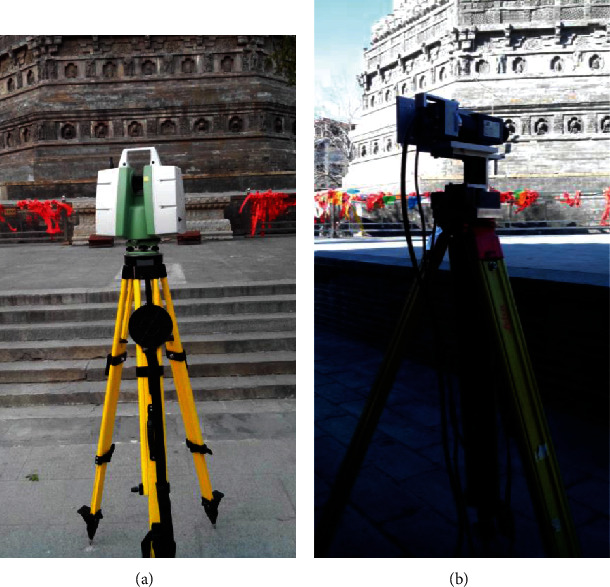
Data acquisition equipment.

The laser scanning system used is Leica Scanstation C10, as shown in Figure 1(a). This scanner is based in the principle of flight of time and has a laser source that emits pulses with a wavelength of 532 nm. The maximal scanning rate is 50,000 points per second. The full scanning field angle is 360° × 270°. The signal intensity received by the sensor system is recorded in [0 255]. The software Leica Cyclone was used for the acquisition and the processing of the data.

The hyperspectral imaging system used is independently integrated by our laboratory, which consists of a hyperspectral camera, a turntable, a controller, and a computer, as shown in Figure 1(b). And the hyperspectral camera parameters are shown in Table 1. The software used for the hyperspectral-data acquisition and processing is also developed by us.

### 2.2. Data Fusion

In this paper, the fusion of laser data and hyperspectral data is to attach hyperspectral Information to point cloud in the same target point. Let laser(*x*_laser_, *y*_laser_, *z*_laser_, *x*_1_, ⋯, *x*_*n*_) be the point cloud information in any target point, where the 3D coordinate of laser is (*x*_laser_, *y*_laser_, *z*_laser_), feature information of laser is (*x*_1_, ⋯, *x*_*n*_). Let hype(*x*_hype_, *y*_hype_, *y*_1_, ⋯, *y*_*m*_) be the hyperspectral information in same target point, where the 2D coordinate of hype is (*x*_hype_, *y*_hype_) and spectral information of hype is (*y*_1_, ⋯, *y*_*m*_). Then, the information of fusion data in this point is laser_hype(*x*_laser_, *y*_laser_, *z*_laser_, *x*_1_, ⋯, *x*_*n*_, *y*_1_, ⋯, *y*_*m*_).

The 3D laser scanning system and the hyperspectral imaging system have the different imaging models, which can generate, respectively, the multidimensional data with three-dimensional coordinates and the multidimensional data with two-dimensional coordinates, and therefore, the fusion between two kinds of data is very difficult. In this paper, “the fusion algorithm based on feature points” is constructed and used. In this algorithm, the point-cloud coordinates are regarded as the corresponding object coordinates of the hyperspectral coordinates, and the mapping relationship between them is constructed. Then, the one-to-one correspondence between point cloud and hyperspectral data is established to complete the registration of the two types of data.

**Figure 2:**
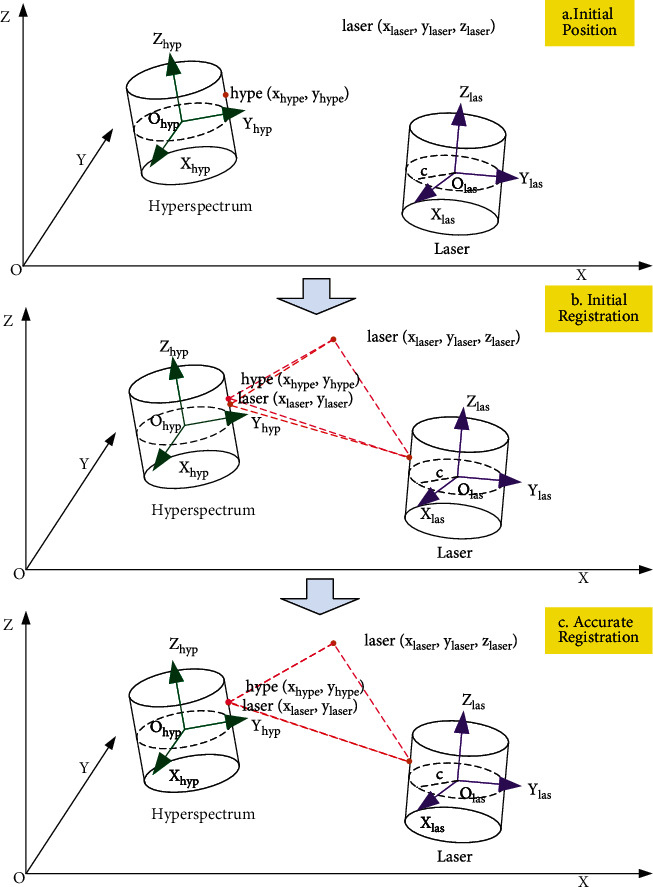
Registration model of laser data and hyperspectral data.

The registration model of the algorithm is as follows: firstly, the corresponding feature points of point cloud and hyperspectral data are selected. According to the laser scanning system and hyperspectral imaging system, the position relation of the same target point is as shown in Figure 2(a), where laser(*x*_laser_, *y*_laser_, *z*_laser_) is the point-cloud coordinate and hype(*x*_hype_, *y*_hype_) is the hyperspectral coordinate. Secondly, through the initial registration by collinear equation, the mapping model between point-cloud coordinate system and hyperspectral coordinate system is built, and laser′(*x*_laser′_, *y*_laser′_) is the corresponding coordinate of laser(*x*_laser_, *y*_laser_, *z*_laser_) generated by the mapping relation. Theoretically, laser′(*x*_laser′_, *y*_laser′_) and hype(*x*_hype_, *y*_hype_) are the same point, but due to errors, there is a little deviation between them, as shown in Figure 2(b). Last distortion correction is used to reduce errors between them, and then the corresponding points of two systems coincide completely, as shown in Figure 2(c). Finally, hype(*y*_1_, ⋯, *y*_*m*_) is attached to laser(*x*_laser_, *y*_laser_, *z*_laser_, *x*_1_, ⋯, *x*_*n*_) by the mapping relation, and laser_hype(*x*_laser_, *y*_laser_, *z*_laser_, *x*_1_, ⋯, *x*_*n*_, *y*_1_, ⋯, *y*_*m*_) is constructed.

The basic steps of the algorithm are as follows. Firstly, the corresponding feature points between point cloud and hyperspectral data are extracted from the points and clouds by our own algorithm. Secondly, according to the collinear equation, the initial correspondence relation is calculated using direct linear transformation. Thirdly, the accurate correspondence is established by the distortion correction through establishing the corrected values and eliminating the errors. Finally, the fusion between them is completed by the parameter of the accurate correspondence relation.

### 2.3. Fusion-Data Detection

**Figure 3:**
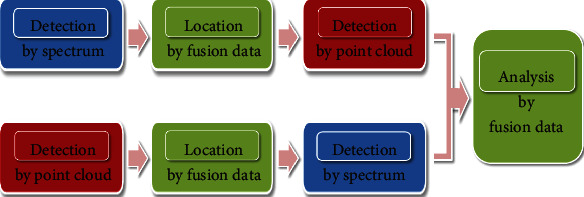
Detecting process of data fusion.

In each case, the detection of the state of conservation and risk of historical buildings usually needs different methods. But generally, its steps may be divided into two categories, as shown in Figure 3.

In the first case, the material changes detect firstly the by the hyperspectral information of hyperspectral data. Then, the corresponding point-cloud regions of the detected-change hyperspectral data are located by the fusion data. Thirdly, the geometrical changes in the corresponding point-cloud region are detected by the geometrical and radiometric information of the corresponding point cloud. Finally, the architectural and constructive characteristics of historical buildings are analyzed thoroughly.

In the other case, it is vice versa, starting from detecting by point-cloud features, then locating the corresponding spectral information by the fusion data, and next detecting by spectral information, up to analyzing for the fusion data.

## 3. Experiment

In order to verify the feasibility and the applicability of this method, the part data of the Tianningsi Tower, as the research object, were selected. The laser data were acquired for the three-dimensional model construction of Tianningsi Tower. The hyperspectral data were collected for this experiment.

### 3.1. Tianningsi Tower

**Figure 4:**
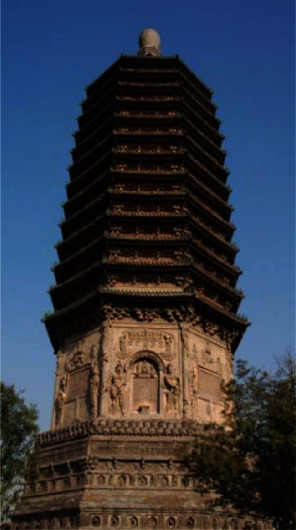
Tianningsi Tower.

Beijing Tianningsi Tower is the oldest and highest building in Beijing (China), initially built from 1119 to 1120, located in Tianning Temple, Guanganmen, Xicheng District, Beijing, as shown in Figure 4. The tower is about 54.87 m high and is an octagonal-plane thirteen-layer solid-brick structure, composed of four parts such as tower-base, tower-body, tower-canopy, and tower-cap. All sides of the tower have many beautiful, well-proportioned brick-sculptures and clay-sculptures like Lotus, Loin, Bodhisattva, Muscleman, and so forth, which form a magnificent work of art and have the strong Buddhist meaning. Tianningsi Tower is one of the most representative Buddhist towers in China, a blending product of Indian Buddhist culture, foreign Western-Region culture, and traditional Chinese Central-Plain culture in the long-term development process of colliding with each other. According to the historical data records, from the prime to the present, the tower has been damaged and repaired several times; moreover, it was influenced by natural conditions. Therefore, the tower height, the building material, and the sculpture modeling have also changed many times. It seems that the tower was initially about 57.8 m high, and it was about 55.38 m high in 1992 [14], but today, it has a height of about 54.87 m under this actual measurement.

### 3.2. Data Acquisition

**Figure 5:**
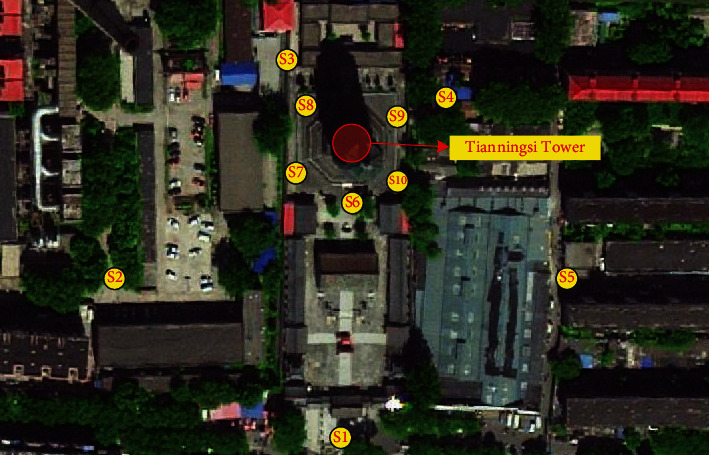
Data acquisition location map of Tianningsi Tower.

Due to fact that Tianningsi Tower is located in the main city of Beijing, where the surrounding buildings are too many and the population is so dense, the acquiring data is more different, as shown in Figure 5. Our Research Group collects laser data and hyperspectral data two times. Laser data was acquired by Leica 3D Laser Scanner, and hyperspectral data was acquired by Sweep Hyperspectral Scanner. According to the tower height and the surrounding environment, the data were acquired by means of ten scans from ten different viewpoints distributed along the adjacent street around the prospect, as shown in Figure 5. Starting from the tower's south part in a clockwise direction, the initial laser scans in the tower upper side are acquired successively by five sites, such as S1, S2, S3, S4, and S5, which contain basic information of the tower's top part. And the initial laser scans in the tower lower side are acquired successively by five sites, such as S6, S7, S8, S9, and S10, which contain basic information of the tower's bottom part. The mean spot spacing was about 3 mm in the main part of the prospect. Moreover, the large overlapping area between adjacent scans can make the total point cloud denser.

**Figure 6:**
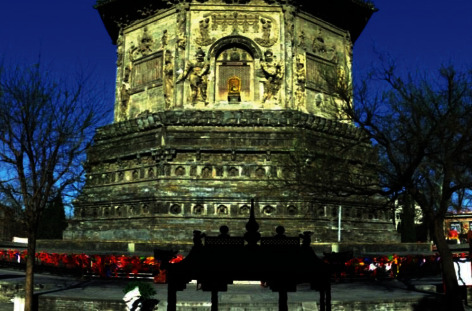
Hyperspectral fusion image in the south of Tianningsi Tower.

Hyperspectral data in the south of Tianningsi Tower were acquired with 420 continuous wave bands and 390–1000 nm wave range. It is shown in Figure 6, which is the fusion image of the band R (191), G (118), and B (44).

### 3.3. Data Processing

**Figure 7:**
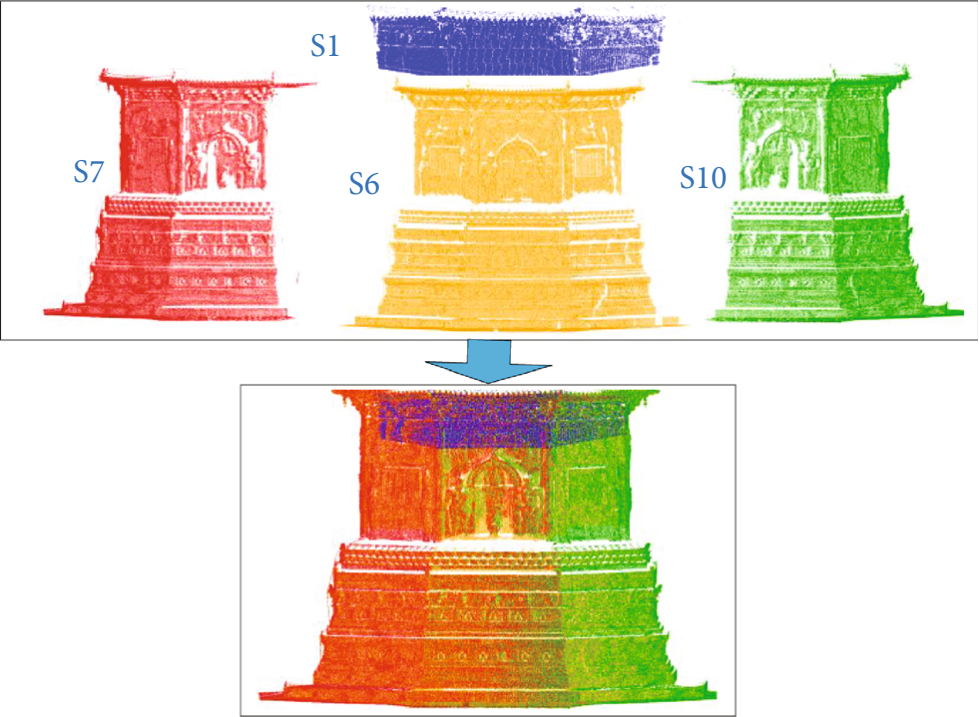
Southern fusion point cloud of Tianningsi Tower.

The fusion experiment is the south part of Tianningsi Tower, according to the collected point cloud, partial data of four sites such as S1, S6, S7, and S10 need to be registered and integrated. In this process, the local registration algorithm uses the registration algorithm based on improved normal distribution transform, which is our previous research method [15]. And the global registration uses an automatic multiscan registration algorithm, which is also our previous research method [16, 17]. The fusion result of point cloud is shown in Figure 7.

Let 3D point-cloud into the 2D gray image, and RGB hyperspectral image into 2D gray image. The feature points of two 2D images are extracted and mapped into three-dimensional space to get the feature points of point cloud and hyperspectral data, and then the six-pair feature points are extracted, as shown in Table 2.

**Figure 8:**
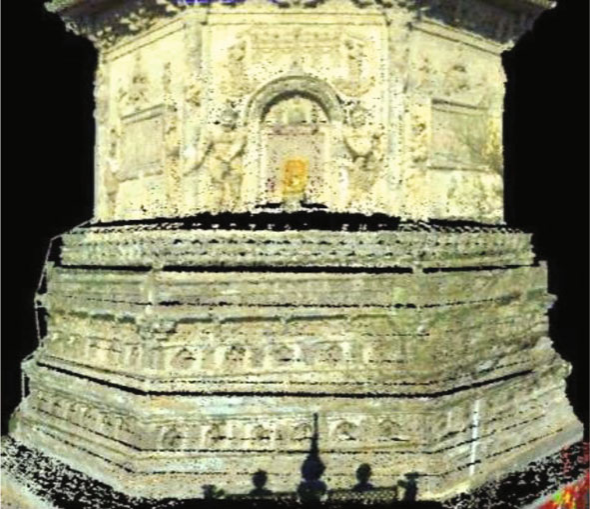
Southern fusion data of Tianningsi Tower.

According to the six-pair feature points, the fusion data of point cloud and hyperspectral data are calculated using the fusion algorithm in above, as shown in Figure 8. Hyperspectral information of Tianningsi Tower is attached to the point cloud information; namely, the fusion data is the point cloud with the spectral information. If *P* is a point, at any point of Tianningsi fusion data, *P* can be expressed as *P*(*x*, *y*, *z*, *m*_1_, ⋯, *m*_*k*_, *n*_1_, ⋯, *n*_420_) containing three kinds of information, where *P*(*x*, *y*, *z*) is the three-dimensional coordinate of fusion data, *P*(*m*_1_, ⋯, *m*_*k*_) is the point-cloud attribute, and *P*(*n*_1_, ⋯, *n*_420_) is hyperspectral feature 8.

## 4. Discussion

**Figure 9:**
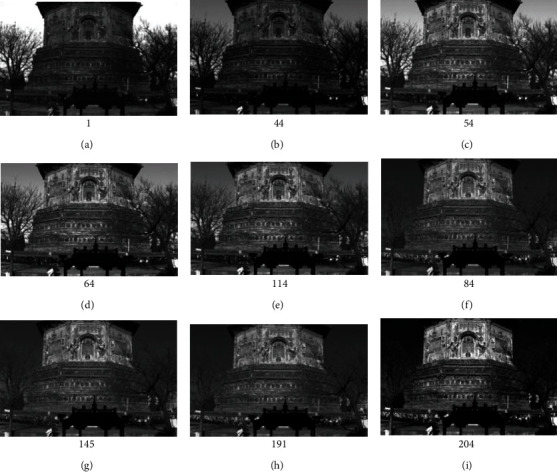
Single band hyperspectral image of Tianningsi Tower.

Hyperspectral data contain hundreds or even thousands of spectral information, which can detect a lot of information undetected by visible light. Twelve single-band hyperspectral images are selected, in which there are some strange bright regions, as shown in Table 3 and Figure 9.

**Figure 10:**
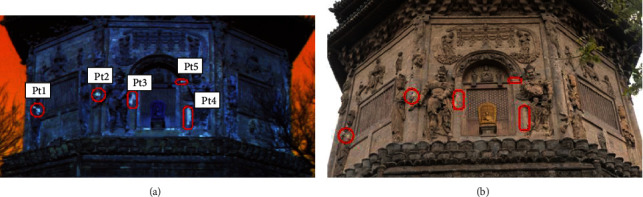
Comparison of false-color high spectral image and digital image.

In order to make the highlighting regions more obvious, the three band images, 1, 6, and 204, which are selected from the tower-body, fused a false-color image, as shown in Figure 10(a). Then, five obvious feature regions are selected, labeled Pt1, Pt2, Pt3, Pt4, and Pt5, respectively, as shown in the red circle section of Figure 10(a). The same areas in digital image are no different with the surrounding areas, as shown in the red circle section of Figure 10(b). After field visiting, looking up historical data, and consulting experts, the highlight areas are the suspected repair-areas. Through hundreds of years of wind and sleet, Tianningsi Tower has been damaged, especially with serious clay damage, and it has been repaired many times. In view of the different repair materials in different periods and the material changes affected by weathering erosion, even if they have the same appearance, the material compositions have great difference.

**Figure 11:**
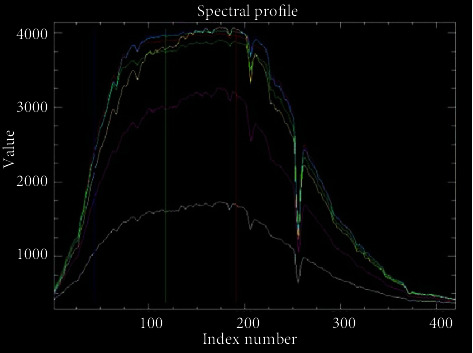
Seven regional DN value spectra in Tianningsi Tower.

In order to further make their difference, integral smoothing DN value spectra of seven areas, including five regions, one clay sculpture region, and one brick sculpture region, are analyzed, and the results are shown in Table 4 and Figure 11. The spectra of five regions, such as Pt1, Pt2, Pt3, Pt4, and Pt5, respectively, have the similar numerical waveforms. But the spectra of S1 and S2 have the great difference from the above five regions, which express clay sculpture region and brick sculpture region in the tower body.

This can be judged, although the appearance of the five regions and their surrounding sculptures looks so alike; in fact, the materials of the five areas may be the same, different from the surrounding. By the historical investigation and the temple inscription records, Tianningsi Tower has undergone many renovations, including several major overhauls, for example, in 1756 and 1782 of the Qing Dynasty, in 1937 and 1941–1943 of the Republic of China, and in 1991–1992 and 2002 of the People's Republic of China. Although the repair materials are still mixed with mud and sand, however, times of the production process and ingredients are different. Furthermore, weathering and erosion also have impact on the composition of the material. Therefore, it is again concluded that the five area materials belong to the same material and that the patches were highly likely available for the same period.

**Figure 12:**
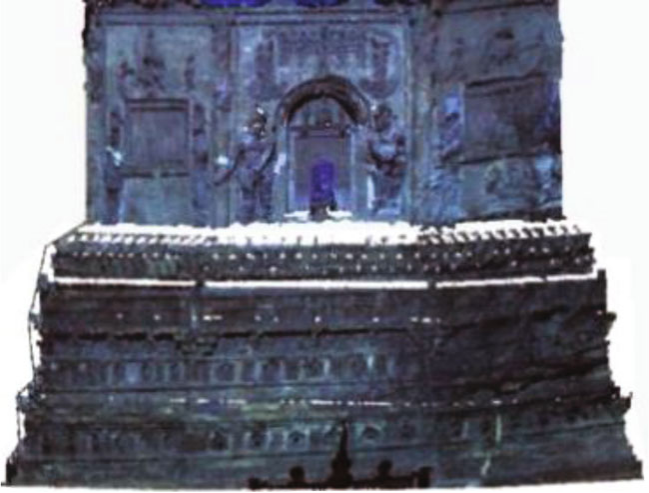
Fusion data of Tianningsi Tower.

As mentioned above, the hyperspectral data can detect the potential spectral feature information to achieve damage detection, age judgment, and information recovery of ancient buildings caused by the different chemical composition, but not judged caused by the different geometric appearance. However, their fusion can also detect the geometric appearance to accurately determine the location of damage-area and repair-area. Therefore, their fusion can detect changes caused by material composition and geometric shape. The fusion data of Tianningsi Tower is shown in Figure 12, in which the repair areas of fusion data have obvious characteristics.

**Figure 13:**
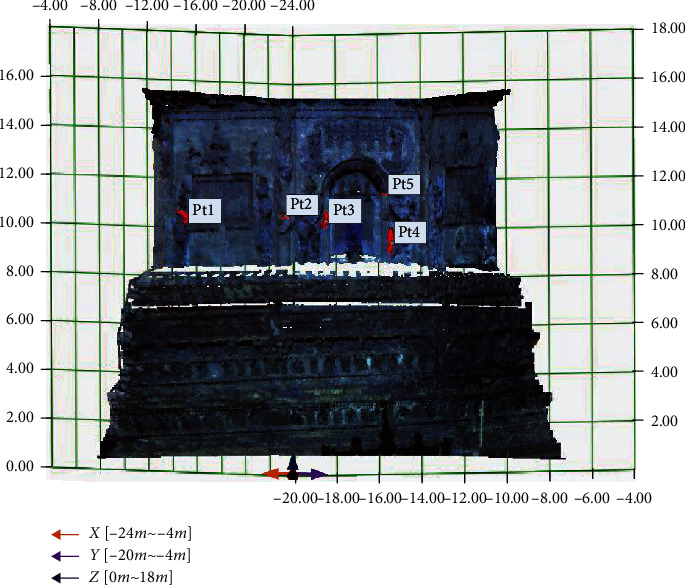
Fusion data of Tianningsi Tower in the grid coordinate.

To further gain the repair-area information like location, shape, and size, according to the point-cloud coordinates, the grid coordinate is constructed, in which the fusion data is shown, where *x*-axis range is [−24 m, −4 m], *y*-axis range is [−20 m, −4 m], and *z*-axis range is [0 m, 18 m]; the grid size is 2 m, as shown in Figure 13. The red parts are the highlighted area detected by the hyperspectra, which have given the point cloud by fusion data. If the intermediate-point coordinates of the repair regions are regarded as the repair-region coordinates, the repair-region location relative to the coordinate system can be determined, as shown in Table 5.

**Figure 14:**
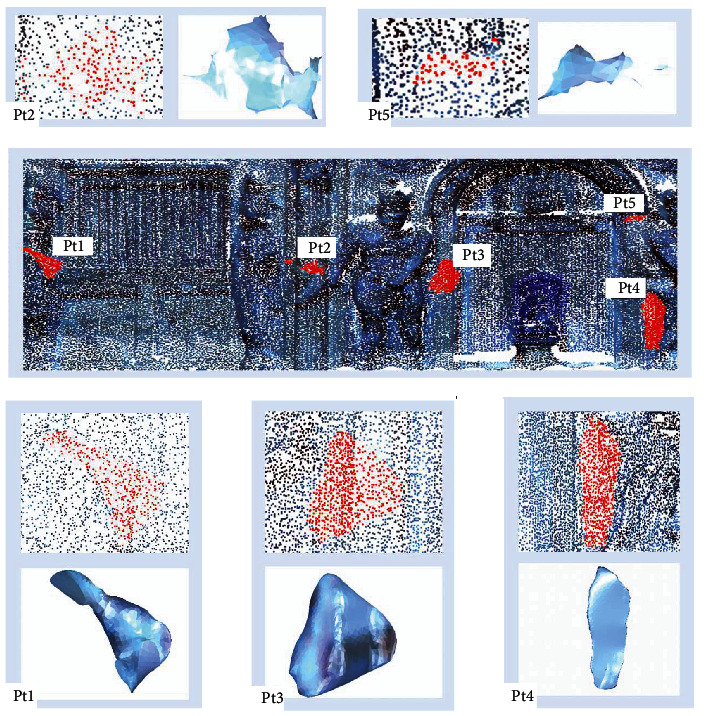
Suspected repair-region shape of Tianningsi Tower.

Moreover, according to the highlight-region point clouds in fusion data, the Triangular mesh surfaces are constructed, whose shape is the repair-region shape and whose area is the repair-region area, as shown in Table 6 and Figure 14.

## 5. Conclusion

This paper reported the detected change methodology using terrestrial laser scanning and hyperspectral imaging in the conservation work on ancient buildings and attributed to Beijing Tianningsi Tower (17th century), China, the famous Buddhist pagoda. Firstly, laser data and hyperspectral data were acquired not on the same time or place. Secondly, two kinds of data were processed, respectively, and then they were registered and fused. Finally, the fusion data were applied to detect changes of historical buildings.

It is possible to assess that the fusion data of laser scanning and hyperspectral imaging offer an effective noncontact method to detect the surface changes without damaging the building. This multifusion, appropriately tested and verified, is an extremely precise and controlled method to detect geometrical and material changes in a selective and highly controlled modality.

All working steps were carefully documented by a multisource data detecting system that allowed for obtaining a comprehensive analysis on which all information gathered during the conservative procedure was inserted. In the field of conservation, the advantages to report the information in a single file linked to the object that, when necessary, can be used to update the more complete and accurate laser data and hyperspectral data, represent a valuable tool for documentation.

## Figures and Tables

**Table 1:** Hyperspectral camera parameters.

Name	Parameter	Name	Parameter
Spectrum range	400-1000 nm	Spectrometer entrance slit width	30 *μ*m
Spectral resolution	2.8 nm	Spectrometer entrance slit length	11.84 mm
FOV	27.2^o^	Camera lens focal length	23 mm
Spatial resolution	1600 pixels	Spectral band numbers	840

**Table 2:** Feature points of point cloud and hyperspectral data.

Hyperspectral data	Point cloud	Hyperspectral data	Point cloud
497, 29	-9.454, -11.619, 14.235	647, 169	-11.969, -9.519, 10.899
727, 32	-13.019, -8.228, 14.208	610, 74	-11.007, -9.727, 13.009
476, 265	-9.079, -11.743, 8.333	743, 273	-13.045, -7.953, 8.370

**Table 3:** Single band hyperspectral image of Tianningsi Tower.

Wave band	1	44	54	64	84	114	145	191	204
Wavelength (nm)	390	449	463	477	505	548	592	660	679

**Table 4:** Correspondence of regions and DN value in Tianningsi Tower.

Area number	Pt1	Pt2	Pt3	Pt4	Pt5	S1	S2
Spectral color	Red	Green	Blue	Yellow	Navy	Purple	White

**Table 5:** Suspected repair-region position of Tianningsi Tower (unit: m).

Suspected repair-region number	Suspected repair-region coordinate	Suspected repair-region position
Pt1	-8.880, -16.523, 10.258	16.880, 4.523, 10.258
Pt2	-8.889, -11.833, 10.198	16.889, 9.833, 10.198
Pt3	-10.255, -10.750, 10.060	14.255, 10.75, 10.060
Pt4	-12.302, -8.800, 9.343	12.302, 12.800, 9.343
Pt5	-12.129, -8.969, 11.147	12.127, 12.969, 11.147

**Table 6:** Suspected repair-region area of Tianningsi Tower (unit: m^2^).

Suspected repair-region number	Pt1	Pt2	Pt3	Pt4	Pt5
Suspected repair-region area	0.167	0.0433	0.234	0.324	0.013

## Data Availability

The datasets used and/or analyzed during the current study are available from the corresponding author on reasonable request.
